# In-hospital mortality after prehospital endotracheal intubation versus alternative methods of airway management in trauma patients. A cohort study from the TraumaRegister DGU®

**DOI:** 10.1007/s00068-024-02498-8

**Published:** 2024-03-20

**Authors:** Moritz Weigeldt, Stefan Schulz-Drost, Dirk Stengel, Rolf Lefering, Sascha Treskatsch, Christian Berger, the TraumaRegister DGU

**Affiliations:** 1 Department of Anesthesiology and Intensive Care Medicine, Charité - Universitätsmedizin Berlin, Corporate Member of Freie Universität Berlin, Humboldt-Universität Zu Berlin, Campus Benjamin Franklin, Hindenburgdamm 30, 12203 Berlin, Germany; 2 Department of Trauma Surgery, Helios Kliniken Schwerin, Schwerin, Germany; 3 BG Kliniken - Hospital Group of the German Federal Statutory Accident Insurance, Leipziger Platz 1, 10117 Berlin, Germany; 4 Institute for Research in Operative Medicine (IFOM), Faculty of Health, Witten/Herdecke University, 51109 Cologne, Germany; 5 Committee On Emergency Medicine, Intensive Care and Trauma Management (Sektion NIS) of the German Trauma Society (DGU), Berlin, Germany

**Keywords:** Preclinical, Airway management, Multiple trauma

## Abstract

**Purpose:**

Prehospital airway management in trauma is a key component of care and is associated with particular risks. Endotracheal intubation (ETI) is the gold standard, while extraglottic airway devices (EGAs) are recommended alternatives. There is limited evidence comparing their effectiveness. In this retrospective analysis from the TraumaRegister DGU®, we compared ETI with EGA in prehospital airway management regarding in-hospital mortality in patients with trauma.

**Methods:**

We included cases only from German hospitals with a minimum Abbreviated Injury Scale score ≥ 2 and age ≥ 16 years. All patients without prehospital airway protection were excluded. We performed a multivariate logistic regression to adjust with the outcome measure of hospital mortality.

**Results:**

We included *n* = 10,408 cases of whom 92.5% received ETI and 7.5% EGA. The mean injury severity score was higher in the ETI group (28.8 ± 14.2) than in the EGA group (26.3 ± 14.2), and in-hospital mortality was comparable: ETI 33.0%; EGA 30.7% (27.5 to 33.9). After conducting logistic regression, the odds ratio for mortality in the ETI group was 1.091 (0.87 to 1.37). The standardized mortality ratio was 1.04 (1.01 to 1.07) in the ETI group and 1.1 (1.02 to 1.26) in the EGA group.

**Conclusions:**

There was no significant difference in mortality rates between the use of ETI or EGA, or the ratio of expected versus observed mortality when using ETI.

## Introduction

Airway management is a central component of major trauma care within the framework of the ABC approach of all current international recommendations [1–3]. The German S3 trauma guideline for the treatment of polytrauma and severely injured patients proposed indications for prehospital emergency anaesthesia and airway management and, regarding other European and international guidelines, endotracheal intubation (ETI) was defined as the gold standard [4–6]. However, prehospital emergency anaesthesia is more demanding and associated with a higher incidence of difficult airway (e.g. haemorrhage, spine protection, environment, lack of expertise) [7–9]. This is even more true for trauma care, where immobilisation of the cervical spine may additively increase the risk of a difficult airway [5, 7, 10]. Physicians’ experience performing ETI is, however, not always sufficient in this situation [11–13] and unrecognized failed ETI often has serious consequences [8, 12, 14].

Extraglottic airway devices (EGAs) are available as an alternative to ventilate patients, especially when ETI fails or is considered difficult [6, 13, 15]. EGAs in emergency medical services include the laryngeal tube, cuffed laryngeal mask, and i-gel laryngeal mask [16] Emergency physicians with little experience in ETI are even advised to primarily use EGAs [7, 11, 17, 18]. However, this technique also requires regular training to ensure proper positioning and function [13, 15, 19].

Complications such as aspiration, leakage, and dislocation are more associated with EGA than ETI [8, 17, 20]. In addition, gastric distention due to improper EGA placement can occur, which can render ventilation ineffective [8, 20, 21]. Though second-generation EGAs have a channel through which gastric contents can be drained [15], this may not always prevent over-inflation [21, 22]. Other EGA-associated potential complications include tongue swelling with subsequent airway obstruction and intubation difficulty, cuff herniation, soft tissue injury, and bleeding [8].

The evidence for the use of EGAs in out-of-hospital cardiac arrest is inconclusive. Some of the literature suggests that they have a negative impact on outcome [13, 20, 23], whereas more recent studies consider the role of EGAs to be equivalent [24, 25]. However, these results have limited transferability into trauma care due to specific pathophysiological differences (cervical spine, haemorrhage, head and neck injuries). In the context of trauma care, a retrospective study from a US level I trauma centre showed no difference in mortality between ETI (with and without rapid sequence intubation [RSI]) and EGA [26]. Another US study also found no difference in the incidence of ventilator-associated pneumonia depending on different airway devices [14]. Both studies, however, were monocentric, and prehospital trauma management, including airway management, was performed by paramedics.

Evidence on outcomes of prehospital trauma airway care is limited, and complicated by differences in healthcare systems and strategies. This retrospective registry query aims to determine if trauma patients treated prehospital with EGA in the German system have higher in-hospital mortality compared to those treated with ETI.

## Methods

A registry evaluation of the TraumaRegister DGU® of the German Trauma Society (DGU) from 01/2015 to 12/2018 was performed.

The TraumaRegister DGU® of the German Trauma Society (Deutsche Gesellschaft für Unfallchirurgie, DGU) was founded in 1993. The aim of this multicentre database is a pseudonymised and standardised documentation of severely injured patients.

Data are collected prospectively in four consecutive time phases from the site of the accident until discharge from hospital: (A) pre-hospital phase, (B) emergency room and initial surgery, (C) intensive care unit, and (D) discharge. The documentation includes detailed information on demographics, injury pattern, comorbidities, pre- and in-hospital management, course on intensive care unit, and relevant laboratory findings including data on transfusion and outcome of each individual. The inclusion criterion is admission to hospital via emergency room with subsequent ICU/ICM care or hospital arrival with vital signs and death before admission to ICU.

The infrastructure for documentation, data management, and data analysis is provided by AUC—Academy for Trauma Surgery (AUC—Akademie der Unfallchirurgie GmbH), a company affiliated to the German Trauma Society. The scientific leadership is provided by the Committee on Emergency Medicine, Intensive Care and Trauma Management (Sektion NIS) of the German Trauma Society. The participating hospitals submit their data pseudonymised into a central database via a web-based application. Scientific data analysis is approved according to a peer review procedure laid down in the publication guidelines of TraumaRegister DGU®.

The participating hospitals are primarily located in Germany (90%), but a rising number of hospitals of other countries contribute data as well (at the moment from Austria, Belgium, Finland, Luxembourg, Slovenia, Switzerland, The Netherlands, and the United Arab Emirates). Currently, almost 30,000 cases from nearly 700 hospitals are entered into the database per year.

Participation in TraumaRegister DGU® is voluntary. For hospitals associated with TraumaNetzwerk DGU®, however, the entry of at least a basic dataset is obligatory for reasons of quality assurance.

This study has been written according to the publication guidelines of TR-DGU and registered under Project ID 2018–021.

**Fig. 1:**
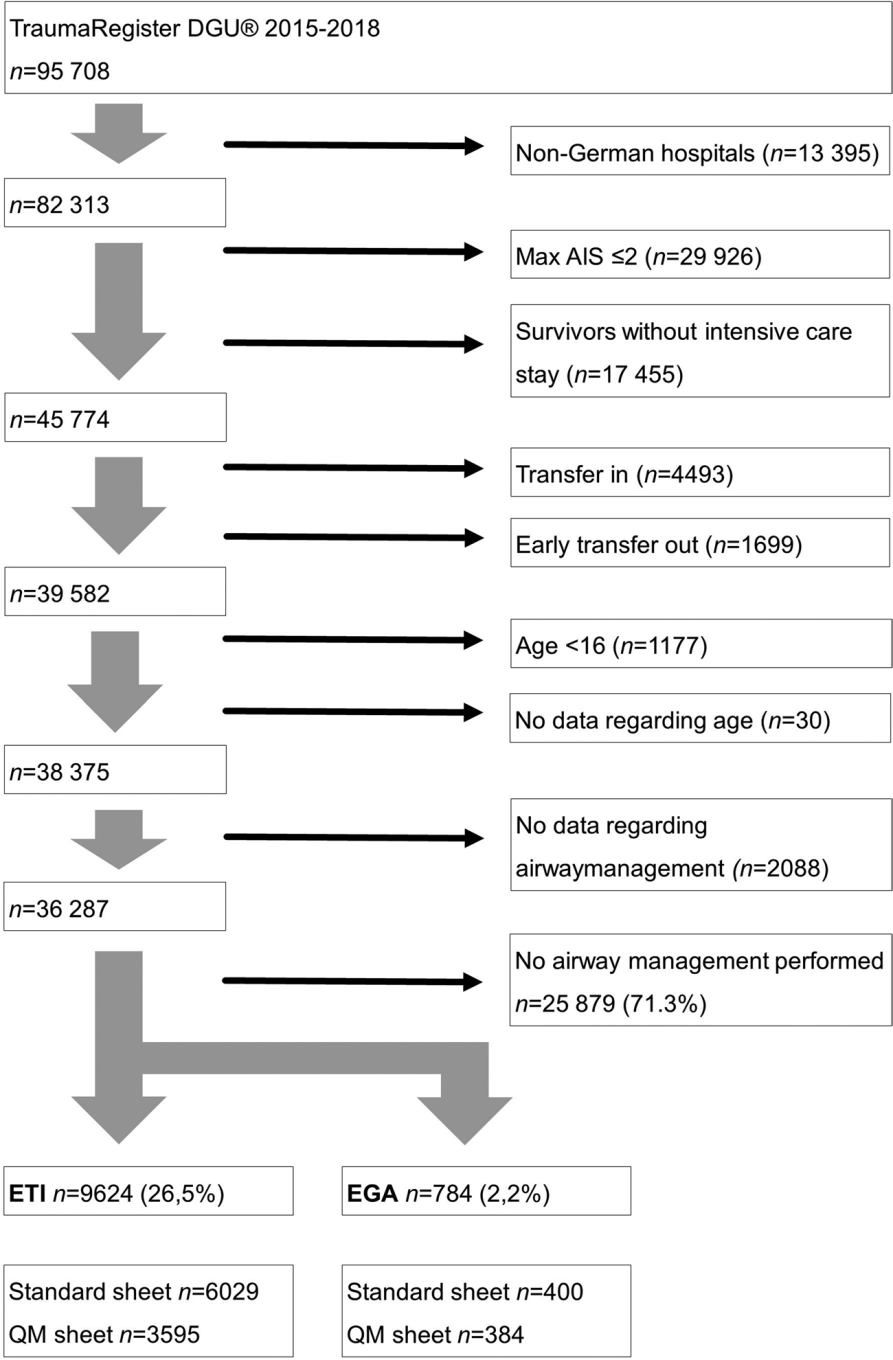
Flow diagram case selection and the selection process

Datasets included a total of *n* = 95,708 patients who were treated in German hospitals from 2015 to 2017 and recorded accordingly in the TR-DGU database. The data entry sheet of TraumaRegister DGU® is available in two versions: The standard sheet was developed when establishing the registry and requires the entry of about 100 parameters. Especially for TraumaNetzwerk DGU®, a shortened version with about 40 parameters, the so-called QM sheet, is existing. Both the standard and the quality management (QM) sheet were used for this analysis [27]. Only cases from German hospitals were included. Cases with a maximum Abbreviated Injury Scale (AIS) score ≤ 2 without an intensive care unit stay or with a secondary transfer were excluded. Age was restricted to ≥ 16 years. All patients without prehospital airway protection were excluded. The airway is only documented according to the criterion of alternative airway management (EGA group) or endotracheal intubation (ETI group); there is no differentiation between the various alternative EGAs. Only the surgical airway is documented separately and is not part of this evaluation (Fig. 1). The clinical endpoint is in-hospital mortality, secondary endpoints included observed vs. expected in-hospital mortality according to the RISC II score, injury pattern according to body region, rescue time, and length of stay in the intensive care unit (ICU) and hospital.

### Statistical analysis

Continuous data were reported as means ± SD, or in case of a skewed distribution, as median [IQR], and categorical variables as numbers with percentages. Differences between the two groups were assessed with Fisher’s exact test, or with Mann–Whitney *U* test. A *P* < 0.05 was considered statistically significant. Due to the large sample size, formal statistical tests were applied in selected comparisons only; differences of 2–3% (categorical variables), or 0.07 SD (metric data), would be statistically significant.

Multivariate logistic regression analysis with hospital mortality as a dependent variable was performed for the following predictor variables: age (4 groups), sex, pre-existing disease (ASA 3/4), injury severity score (ISS), location of relevant injury (head, thorax, abdomen, extremities/pelvis, with AIS ≥ 3), unconsciousness (GCS ≤ 8), prehospital shock (systolic blood pressure ≤ 90 mmHg), prehospital interventions (chest tube, catecholamine administration, pelvic binder, cardiac arrest with resuscitation), transport by helicopter or ground-based, darkness (dark hours defined per month), and level of care of the receiving hospital. The results of logistic regression analysis are presented as odds ratio (95% confidence intervals). Secondary endpoints included observed vs. expected mortality according to the RISC II score, with a standardized mortality ratio and 95% CI. Statistical analysis was performed using SPSS, version 25.0 (IBM Inc., Armonk, NY, USA).

## Results

There were *n* = 10,408 cases included in the analysis, of which *n* = 9624 cases (92.5%) received endotracheal intubation (ETI group) and *n* = 784 cases (7.5%) received EGA management (EGA group). A higher proportion of men was found in both groups, and patients were slightly older in the EGA group (Table 1). A blunt accident mechanism was the main cause in both groups, but the proportion was greater in the EGA group. The majority of causes were traffic accidents and falls. More patients were intubated in air-transported patients; however, EGA was performed more frequently in ground-transported patients as well as during darkness.

**Table 1:** Basic parameters, course of trauma, and mode of transport

Demographics	ETI *n* = 9624 (92.5%)	EGA *n* = 784 (7.5%)	Total *n* = 10,408
Male sex, (*n*))	6831 (71.0%)	565 (72.1%)	7396 (71.1%)
Age (years), mean (*SD*)	52 (22)	54 (21)	53 (22)
Age ≥ 70, (*n*)	2567 (26.7%)	218 (27.8%)	2785 (26.8%)
Trauma mechanism			
Blunt trauma, (*n*)	8719 (85.2%)	718 (94.2%)	9437 (95.1%)
Road traffic accident, (*n*)	5101 (53.6%)	(398 (51.3%)	5499 (53.4%)
Car/lorry, (*n*)	2258 (23.7%)	154 (19.8%)	2412 (23.4%)
Motorcycle, (*n*)	1122 (11.8%)	93 (12.0%)	1215 (11.8%)
Bicycle, (*n*)	791 (8.3%)	71 (9.1%)	862 (8.4%)
Pedestrian, (*n*)	729 (7.7%)	66 (8.5%)	795 (7.7%)
Others, (*n*)	201 (2.1%)	14 (1.8%)	215 (2.1%)
Fall > 3 m, (*n*)	1457 (15.3%)	136 (17.5%)	1593 (15.5%)
Fall < 3 m, (*n*)	2032 (21.4%)	180 (23.2%)	2212 (21.5%)
Assault, (*n*)	289 (3.0%)	15 (1.9%)	304 (3.0%)
Gunshot, (*n*)	129 (1.4%)	5 (0.6%)	134 (1.3%)
Stabbing, (*n*)	157 (1.7%)	21 (2.7%)	178 (1.7%)
Other, (*n*)	349 (3.7%)	21 (2.7%)	370 (3.6%)
Transportation			
During darkness, (*n*)	3412 (35.7%)	332 (42.7%)	3744 (36.2%)
Transport by emergency physician	9250 (99.6%)	745 (97.9%)	9995 (99.5%)
Transport by helicopter emergency medical service (HEMS), (*n*)	3700 (39.9%)	121 (15.9%)	3821 (38.0%)

### Injury severity and pattern

Patients in the ETI group were more severely injured, and systolic blood pressure was more frequently below 90 mmHg. In addition, the incidence of traumatic brain injuries (TBI) was higher and associated with lower initial GCS values (see Table 2).

**Table 2:** Injury severity and pattern

Injury severity	ETI	EGA	Total
Injury Severity Score (ISS), median [IQR]	25 [18 to 34]	24 [16 to 34]	25 [17 to 34]
New ISS, median [IQR]	34 [24 to 48]	29 [22 to 42]	34 [22 to 48]
Relevant injuries (AIS ≥ 3) in different body regions			
Head, (*n*)	6389 (66.4%)	440 (56.1%)	6829 (65.6%)
Thorax, (*n*)	4882 (50.7%)	415 (52.9%)	5297 (50.9%)
Abdomen, (*n*)	1222 (12.7%)	98 (12.5%)	1320 (12.7%)
Extremities/pelvis, (*n*)	3049 (31.7%)	238 (30.4%)	3287 (31.6%)
Face, (*n*)	898 (9.3%)	53 (6.8%)	1870 (9.1%)
Traumatic brain injury (TBI) combined with other injuries, (*n*)	5226 (54.3%)	367 (46.8%)	5593 (53.7%)
Isolated TBI, (*n*)	1901 (19.8%)	137 (17.5%)	2038 (19.6%)
GCS < 14, (*n*)	7446 (80.8%)	503 (65.5%)	7949 (79.6%)
GCS 3–8, (*n*)	5786 (62.8%)	336 (44.3%)	6122 (61.4%)
Systolic blood pressure (SBP) (mmHg) SBP ≤ 90 mmHg, (*n*)	1779 (21.2%)	119 (17.1%)	1898 (20.9%)

### Pre-hospital interventions and vital signs

In the ETI group, the need to infuse a large amount of volume pre-hospital was increased, sedation was induced more often, and resuscitative measures were intensified (Table 3). Rescue times were longer as well as for time from accident to hospital (ETI 73 min vs. EGA 63 min; median) and for on-scene time (ETI 35 min vs. EGA 27 min; median) in the ETI group.

**Table 3:** Pre-hospital interventions and initial vital signs

	ETI	EGA	Total
Volume			
≤ 500 ml, mean	2923 (33.2%)	361 (49.7%)	3284 (34.5%)
501–1000 ml, mean	2947 (33.5%)	212 (29.2%)	3159 (33.2%)
1001–2000 ml, mean	2330 (26.5%)	132 (18.2%)	2462 (25.8%)
> 2000 ml, mean	600 (6.8%)	21 (2.9%)	621 (6.5%)
Tranexamic acid, (*n*)	1446 (15.5%)	56 (7.4%)	1502 (14.9%)
Resuscitation, (*n*)	1243 (12.9%)	100 (12.8%)	1343 (12.9%)
Pelvic binder*, (*n*)	894 (14.8%)	41 (10.3%)	935 (14.5%)
Catecholamines*, (*n*)	1806 (30.0%)	66 (16.5%)	1872 (29.1%)
Chest tubes*, (*n*)	655 (10.9%)	11 (2.8%)	666 (10.4%)
Sedation*, (*n*)	5095 (84.5%)	273 (68.3%)	5368 (83.5%)
SBP (mmHg), mean (SD)	120 (45)	126 (44)	121 (45)
Heart rate (B/min), mean (SD)	92 (30)	88 (29)	91 (30)
Respiratory rate (RR) (B/min), mean (SD)	14.3 (7.6)	14.8 (8.9)	14.3 (7.7)

^*^Variables are recorded only in the standard sheet (*n* = 6429)

### Clinical treatment

93.1% of patients in the ETI group and 89.7% in the EGA group were directly transferred from the trauma room to the ICU. In the ETI group, patients remained intubated longer and stayed longer in the ICU and hospital. The oxygenation index (Horowitz index) was comparable between groups (*P* = 0.3, Table 4). Most patients were treated in a level 1 trauma centre, only 17.1% of all patients in a level 2 respectively and 2.4% in a level 3 trauma centre.

**Table 4:** Clinical treatment in patients with ICU admission

Patients with ICU admission	ETI	EGA	Total	*P* value
	*n* = 8963	*n* = 703	*n* = 9666	
Days intubated, median [IQR]	2 [1 to 10]	1 [0 to 6]	2 [1 to 10]	0.001
Days on ICU, median [IQR]	6 [2 to 17]	3 [1 to 12]	6 [2 to 17]	< 0.001
Days in hospital, median [IQR]	15 [4 to 28]	13 [4 to 26]	15 [4 to 27]	0.029
PaO_2_/FiO_2_ ratio*, median [IQR]	161 [79 to 300]	152 [77 to 216]	160 [79 to 300]	0.30

^*^Variables are recorded only in the standard sheet (*n* = 6429)

### Mortality and expected mortality

In-hospital mortality was comparable between groups. However, the observed in-hospital mortality was lower than the expected mortality according to the calculated Revised Injury Severity Classification, version 2 (RISC II) [28, 29] in the ETI group (Table 5).

**Table 5:** Mortality and prognosis (numbers)

Mortality	ETI	EGA	Total	*P* value
Died within 24 h, (*n*)	1746 (18.1%)	154 (19.6%)	1900 (18.3%)	0.29
Died in hospital, (*n*)	3175 (33.0%)	241 (30.7%)	3416 (32.8%)	0.20
Risk of death based on RISC II	31.8%	26.9%	31.5%	< 0.001
Standardized mortality ratio (observed/expected)	1.04 (1.01 to 1.07)	1.10 (1.02 to 1.26)	1.04 (1.01 to 1.07)	0.058

Multivariate logistic regression was performed to adjust with the outcome measure of hospital mortality, taking into account known prognosis-relevant factors (Table 6).

**Table 6:** Multivariate logistic regression analysis with hospital mortality as a dependent variable

	Regression coefficient *b*	Odds ratio (95% confidence intervals)	*P* value
Level of care (reference: level I)			0.014
Level II	0.225	1.25 (1.06 to 1.47)	0.006
Level III	0.295	1.34 (0.90 to 2.01)	0.149
Transportation by HEMS	− 0.345	0.71 (0.62 to 0.81)	< 0.001
Darkness	− 0.176	0.84 (0.74 to 0.95)	0.006
ISS (per point)	0.072	1.07 (1.07 to 1.08)	< 0.001
AIS head ≥ 3	0.010	1.01 (0.85 to 1.20)	0.911
AIS thorax ≥ 3	− 0.952	0.39 (0.33 to 0.45)	< 0.001
AIS abdomen ≥ 3	− 0.028	0.97 (0.80 to 1.18)	0.778
AIS extremities ≥ 3	− 0.600	0.55 (0.47 to 0.64)	< 0.001
Chest tube*	0.199	1.22 (0.94 to 1.58)	0.133
Catecholamines*	0.348	1.42 (1.20 to 1.67)	< 0.001
Pelvic binder*	− 0.173	0.84 (0.67 to 1.06)	0.142
Male patient	− 0.018	0.98 (0.87 to 1.11)	0.774
Age (reference: < 60 years)			< 0.001
60–69 years	0.644	1.91 (1.61 to 2.26)	< 0.001
70–79 years	1.362	3.91 (3.32 to 4.60)	< 0.001
80 + years	2.426	11.32 (9.40 to 13.62)	< 0.001
GCS ≤ 8 prehospital	1.191	3.29 (2.84 to 3.81)	< 0.001
Shock (SBP ≤ 90 mmHg)			< 0.001
-Prehospital	0.575	1.78 (1.52 to 2.08)	< 0.001
-On admission	0.429	1.54 (1.28 to 1.85)	< 0.001
ASA 3/4	0.353	1.42 (1.22 to 1.66)	< 0.001
Cardiac arrest prehospital	1.963	7.12 (5.87 to 8.63)	< 0.001
Extraglottic airway	**0.087**	**1.091 (0.87 to 1.37)**	**0.454**
Constant	− 4.271	0.014	< 0.001

^*^Variables are recorded only in the standard sheet

## Discussion

After adjusting for imbalances, we find no difference in in-hospital mortality for prehospital airway management between EGA and ETI. To our knowledge, this is the first study examining these questions within a multicentre register approach in a physician-based rescue service system in Germany.

In general, studies comparing ETI and EGA in trauma patients in terms of mortality are rare, and evidence is limited. In most studies, airway management is performed by paramedics, sometimes even without the use of anaesthetic medications [14, 26, 30]. In addition, sometimes no airway device was placed at all and intubation was delayed until hospital admission [14]. Hence, comparability with a physician-based rescue system is difficult. The experience and use of anaesthetic drugs for airway management are crucial for success and highly inhomogeneous in ambulance systems performed by paramedics and even by physicians whose guidelines recommend the use of medications and training [9, 31].

A systematic review addressing different airway management devices found insufficient evidence regarding benefits and harms in trauma patients: only one study was found, and most studies addressed cardiac arrest [32]. The transferability from cardiac arrest scenarios to trauma care may thus be limited. Airway management in cardiac arrest does not need anaesthesia “induction” drugs, and there is a need not to interrupt chest compression. Recommendations state to start with basic airway techniques and progress stepwise until effective ventilation is achieved, ETI is only recommended for experienced personnel with a high success rate [25, 33]. In contrast, in trauma care, “inductive” anaesthesia/sedation is mostly recommended, taking into account compromising haemodynamics as well as aggravating aspiration or soft tissue swelling by existing injuries/bleeding [9, 34]. Also there is a higher probability that EGAs will dislocate while chest compressions are performed [25].

It should also be added that prehospital rescue equipment is not uniform [35]. Also, in the current German S1 guideline prehospital airway management, the use of a specific device is not recommended, but rather the use of a device with which the user is experienced [9].

Indications for emergency anaesthesia according to the German Society of Anaesthesiology and Intensive Care Medicine (DGAI) recommendation for prehospital emergency anaesthesia (respiratory insufficiency, unconsciousness, or the presence of severe trauma with haemodynamic instability, hypoxia, or traumatic brain injury with a GCS < 9) could be confirmed in our study in conjunction with intensified resuscitative measures [5, 34]. In particular, TBI, alone or in combination, and lower GCS are more common in the ETI group. This is in line with many recommendations, although this is controversial in the literature [36, 37]. Another indication for ETI includes the haemodynamically unstable patient, as ETI was more frequent in the subgroup of patients with SBP ≤ 90 mmHg. Interestingly, original respiratory problems seem not to be associated with ETI in trauma patients, as the mean respiratory rate was the same between the groups.

We cannot make a statement regarding the indication for a specific device, or whether it was used due to failed intubation or lack of experience or as primary device. The use of an EGA can be an alternative fallback option in cases of failed ETI, which may be associated with severe illness and/or inability to adequately perform ETI, especially in stressed situations that remains unclear [1–3, 5]. However, this might lead to a higher mortality rate in the EGA group, which could not be demonstrated in our study. Desaturation itself can increase stress levels and time pressure, which is certainly another important factor. However, how a “cannot intubate” situation is handled differently depends on the experience and level of training of the provider and is difficult to distinguish. On the contrary, in a difficult airway situation, the use of EGA by an experienced provider can help to ensure adequate oxygenation/ventilation of the patient and thus decrease stress levels. Nevertheless, the use of EGA could be an indicator of their lack of experience, if the provider is less trained in ETI and airway management. In this respect, the higher proportion of HEMS in the ETI group could be due to the fact that there are more experienced physicians in the ETI group, as HEMS is performed by better trained physicians compared to ground-based EMS. Patients were also more likely to receive a successful ETI if they were transported with an emergency physician. It should be noted that only a very small number of patients were transported without an emergency physician.

In our analysis, we found that patients with relevant injuries to the face (AIS > 2) are more likely to be treated with an ETI. This is consistent with existing literature, which has shown that airway management is more frequently performed in cases involving facial injuries than in those without [38]. However, the presence of relevant thoracic trauma (AIS body region thorax > 2) or respiratory insufficiency (e.g. mean respiratory rate) was not different between the two groups in our analysis.

A discrepancy is seen in our study between the expected and observed in-hospital mortality. In both groups, prognosis was better than the observed mortality without a statistically significant difference between groups. Additionally, it is uncertain whether this slightly unfavourable ratio is of any clinical significance. However, it is noteworthy that patients in the ETI group had a higher severity of injury and a higher rate of TBI, haemodynamic instability, more frequent countermeasures, longer rescue times, and a higher proportion of helicopter transport, which could introduce a bias for a better prognosis in the EGA group. Whether this association is related to the use of EGA remains unclear, but it may be a surrogate for a reduced necessity of intensified care to secure optimal treatment of trauma patients on scene.

In addition, the association of lower observed mortality for higher AIS scores regarding extremity trauma is congruent with existing literature. Although extremity trauma has an impact on long-term outcomes, the initial life-threatening effects can be addressed (for example pressure bandage, tourniquet, and reduction) [39]. Severe chest trauma was also associated with lower mortality, although chest trauma is the third leading cause of death in polytrauma patients. This may be because most patients do not require further surgery; in most cases, thoracic drainage is a definitive treatment rather than a temporary one [40].

Interestingly, darkness was associated with decreased mortality (see Table 6); here, the rate of EGA was significantly increased compared with daylight interventions. The correlation remains unclear as there is no evidence of greater experience in emergency medicine in rescue teams at night in Germany [41–44]. However, a study from Asia was able to demonstrate more severe road traffic injuries and decreased survival during night time, but no difference in basic or advanced airway management was detected [45].

According to the regression analysis, the sole provision of care by air rescue again has a positive effect on mortality. This has already been demonstrated in a study from the TR-DGU 2016. Here, it was shown that more prehospital measures (intubation, chest tubes, vasopressors, sedation, and volume administration) were performed in the context of air rescue [46]. A comparison of air and ground transport from the USA supports these findings for severe injuries (GCS < 9, haemato-/pneumothorax) [47]. This applies to all different rescue service types and longer rescue time could be explained by the increased number of appropriately performed prehospital measures by competent caregivers [48, 49].

Despite the similar injury severity of patients, there was longer intensive care and hospital length of stay as well as duration of mechanical ventilation in the ETI group. This may be explained by the higher rate of TBI, the necessity for weaning from mechanical ventilation itself, and/or intensified medical measures including complications (e.g. ventilation-associated pneumonia). However, though an increased length of stay, a retrospective single-centre study did not find an increased 24-h mortality rate due to medical measures [50].

Anomalies were seen in the recording of sedation in patients in whom respiratory management was performed prehospital. The need and maintenance for emergency anaesthesia are clearly described in the guidelines [5, 34]. However, lack of (documented) sedation occurred significantly more often in the EGA group: *n* = 1061 (16.5%) patients did not receive prehospital sedation. A possible explanation could be resuscitation, in which sedation is not mandatory. Resuscitation was performed in *n* = 1343 (12.9%) of all cases with an equal distribution between both groups.

There may also be a possible relationship between the lack of sedation and the use of an EGA, as intubation conditions are much worse, and thus alternative airway protection may be needed more frequently. Whether this is due to little experience of the rescue team remains speculative. Other possible causes for a lack of sedation could be a documentation deficit or possibly that sedation is only defined as maintenance for anaesthesia and an induction dose is not considered as sedation. The level of care of the admitting hospital was confirmed as a negative predictor for mortality, as were age, ISS, TBI, comorbidities, haemodynamic instability, catecholamine, and cardiac arrest [5, 51–60].

### Limitations

Overall, the total number of EGAs was relatively low, and thus groups were not comparable. In addition, a propensity score matching was not possible. Within the TR-DGU, the indication for airway management was not recorded, as well as whether EGA was chosen as a secondary solution to a failed intubation attempt or as the primary device. Also, the different devices (laryngeal mask, laryngeal tube, combitube) and the availability of videolaryngoscopy were not documented. The level of training and qualification of the emergency physician was also not recorded, nor was the time. This leads to the fact that the stage at which EGA was selected was not assessed; e.g., whether respiratory insufficiency with desaturation was already present, the initial EGA was replaced by a more experienced provider or adequate analgesia was performed. When invasive airway management is performed, (mechanical) ventilation is mostly needed. Again, no data were available from TR-DGU regarding respiratory rate, ventilation pressure, and CO_2_ values, which may have an important impact on outcome. Finally, some prehospital measures (chest drainage, administration of catecholamines, pelvic binder, and sedation) were only recorded in the standard sheet that was not used in all cases, resulting in a lower number of evaluable cases for these interventions.

Rescue times were shorter in the EGA group, but significantly more medical procedures were performed. Current guidelines recommend exchanging EGA for an endotracheal tube in the trauma room [5], which may contribute to a longer length of stay as it is evident for medical measures performed preclinical [48]. The TR-DGU specializes in trauma and its immediate aftermath, so derivative conclusions about specific techniques such as alternative airway management in general may be limited.

Due to the low number of patients with penetrating accident mechanism trauma (total *n* = 482; EGA group *n* = 44), the validity of this entity is severely limited.

## Conclusion

In conclusion, after adjustment for baseline profile imbalances, the risk of death was not significantly different between ETI and EGA.

The differences observed between the groups were a higher rate of traumatic brain injury and haemodynamic instability, more frequent use of medical countermeasures, longer rescue times, and a higher proportion of helicopter transport in the ETI group. The severity of injury was also higher in the ETI group, which could introduce a bias for a better prognosis in the EGA group. Whether this association is related to the use of EGA remains unclear, but it may be a surrogate for a reduced necessity of intensified care to secure optimal treatment of trauma patients on scene.
